# Contemporary Review of the Methods for Rapid Ventricular Pacing During Transcatheter Aortic Valve Replacement

**DOI:** 10.1016/j.shj.2024.100306

**Published:** 2024-06-13

**Authors:** Eliza Berman, Arsalan Abu-Much, Mark Reisman, Nathan E. Matzko, Jose M. Dizon, Bjӧrn Redfors, Maria C. Alu, Tamim M. Nazif, Martin B. Leon, Shmuel Chen

**Affiliations:** a Weill-Cornell Medical Center/New York-Presbyterian Hospital, New York; b University of British Columbia, Vancouver, British Columbia, Canada; c Cardiovascular Research Foundation, New York; d Division of Cardiovascular Medicine, New York-Presbyterian Hospital, Columbia University Irving Medical Center, New York; e Department of Cardiology, Sahlgrenska University Hospital, Gothenburg, Sweden

**Keywords:** LV pacing, rapid pacing, RV pacing, transcatheter aortic valve replacement, TAVR, TAVI, rapid ventricular pacing

## Abstract

Transcatheter aortic valve replacement (TAVR) is a widely accepted treatment strategy for patients with severe aortic stenosis across all risk profiles. Pacing stimulation of the right ventricle (RV) is the conventional method used during TAVR for rapid pacing during balloon dilatation and transcatheter heart valve deployment and for the management of acute bradyarrhythmias. However, RV pacing requires additional venous access and carries a risk of RV perforation and cardiac tamponade. An alternate strategy of utilizing the stiff guidewire in the left ventricle for direct left ventricle pacing during valve deployment is increasingly being adopted, as it may reduce procedure cost, duration, and radiation exposure and potentially mitigate the risks associated with RV pacing. The current review aims to discuss contemporary rapid pacing techniques for TAVR, including their relative safety, efficiency, and outcomes.

## Highlights

• Rapid ventricular pacing is necessary during transcatheter aortic valve replacement and is typically done via stimulation of the right ventricle.
• Left ventricular pacing has similar efficacy to right ventricular pacing, with possible improved safety.
• Large prospective trials are needed to compare the safety and efficacy of these pacing strategies.

## Graphical abstract

**Figure:**
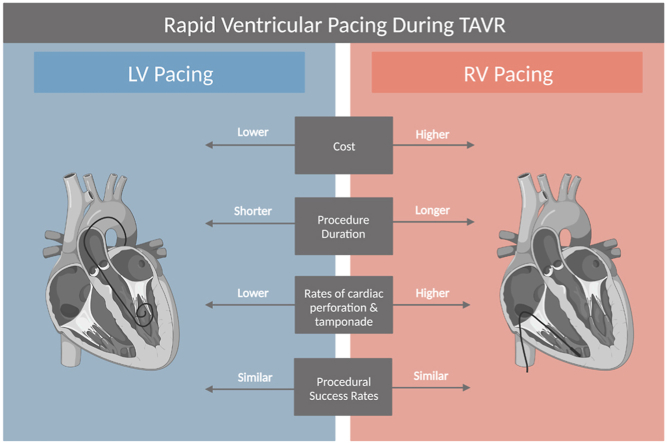

## Introduction

Over the past 2 ​decades, transcatheter aortic valve replacement (TAVR) has become an established treatment for patients with symptomatic, severe aortic stenosis.^1^, ^2^, ^3^, ^4^, ^5^ Refinements in the design of valve prostheses and their delivery systems, new implantation techniques, and increasing operator experience have led to a substantial reduction in TAVR-related adverse events.^6^, ^7^, ^8^, ^9^, ^10^ Furthermore, various measures have been implemented aiming to transform TAVR into a "minimalist" procedure to simplify and shorten the procedure, reduce costs, and improve overall outcomes. Examples include the use of conscious sedation rather than general anesthesia, radial artery for secondary arterial access, and the avoidance of Foley catheters.^11^, ^12^, ^13^, ^14^ As the field continues to evolve, further such refinements are of paramount importance.

Rapid ventricular pacing has been utilized during TAVR to create a temporary reduction in cardiac output in order to facilitate balloon aortic valvuloplasty (BAV), implantation of transcatheter heart valves (THVs), and valve postdilatation. Although the stage at which rapid pacing may be necessary differs between valve types and operators, some form of rapid ventricular pacing is required in most TAVR procedures. In addition, high-degree heart block or other bradyarrhythmias may occur during the procedure, which requires back-up pacing.

Conventionally, rapid ventricular pacing has been carried out using a temporary pacing lead introduced via the jugular or femoral vein with the lead tip placed in the right ventricular (RV) apex. However, RV pacing necessitates venous access and conveys risks related to the access site (bleeding, vascular complication, thrombosis, or infection) and to the placement of the lead in the RV (RV perforation).^15^, ^16^, ^17^, ^18^, ^19^ Rapid pacing through the left ventricular (LV) stiff guidewire has the potential to eliminate these risks while also improving procedural efficiency and has been demonstrated to be a viable technique.^20^, ^21^, ^22^, ^23^, ^24^, ^25^, ^26^, ^27^, ^28^, ^29^ In this article, we review the current state of knowledge regarding the safety and efficacy of rapid pacing with the LV stiff guidewire vs. a temporary transvenous RV lead during transcatheter aortic valve deployment.

## Methods

A systematic literature search for relevant articles published prior to March 2023 was carried out in the PubMed, EMBASE, and Web of Science databases according to the Preferred Reporting Items for Systematic Reviews and Meta-Analyses statement.^30^ Two preliminary reviewers (E.B. and N.M.) screened the searched databases for inclusion. Studies were eligible if they included patients receiving LV pacing during TAVR. Case series with fewer than 20 patients were excluded from the current review, as were studies discussing only RV pacing or rapid pacing for BAV. Studies without mention of complication rates were also excluded. Studies were restricted to those published in English and those conducted on humans. All preliminary screened studies underwent a second round of eligibility screening before investigators independently evaluated and subsequently extracted all relevant data from the selected studies. The extracted data were then organized and reported in the tables. The full Preferred Reporting Items for Systematic Reviews and Meta-Analyses flow chart outlining the study selection process can be found in Supplementary Figure 1. In total, 11 studies were included in the current review. A single randomized trial, EASY TAVI,^23^ prospectively assessed the impact of rapid pacing strategies (LV vs. RV) on total procedure duration and clinical outcomes (Table 1). Five other studies^25^^,^^27^, ^28^, ^29^^,^^43^ retrospectively examined the clinical outcomes of TAVR with direct LV rapid pacing and compared them with those of patients who underwent TAVR with RV pacing (Table 1). The remaining five studies^20^, ^21^, ^22^^,^^24^^,^^31^ evaluated only patients receiving LV pacing to determine its efficacy (Table 1). Table 2 describes the patient characteristics in the included studies.

**Table 1:** Characteristics of the included studies

Study (ref. #)	Study type	*N*			Exclusion criteria
		Total	LV	RV	
Faurie et al., 2019^23^	Prospective, multicenter, randomized	303	151 (49.8%)	152 (50.2%)	Patients undergoing transapical or transaortic procedures
Savvoulidis et al., 2022^27^	Prospective, single center	1226	756 (61.7%)	470 (38.3%)	Patients undergoing TAVR with a planned access route other than femoral or previous enrollment in this or another trial; patients who received a valve other than SAPIEN 3 or XT
Hokken et al., 2021^28^	Retrospective, single center	672	488 (72.6%)	45 (6.7%)	Patients undergoing transapical or transaortic procedures; patients with pre-existing conduction disturbances
Stąpór et al., 2020^25^	Retrospective, single center	143	82 (57.3%)	61 (42.7%)	Patients receiving non-LV pacing (RV-TPW pacing or pacing with PPM)
Kotronias et al., 2019^29^	Retrospective multicenter	529	226 (42.7%)	303 (57.3%)	Patients receiving RV-TPW pacing
Scarsini et al., 2019^31^	Retrospective multicenter	263	263	—	Patients receiving RV-TPW pacing
Spaziano et al., 2017^43^	Prospective, single center	184	142 (77.2%)	42 (22.8%)	Patients with pre-existing conduction disturbances
Hilling-Smith et al., 2017^22^	Prospective, single center	132	132	—	Patients receiving RV-TPW pacing; patients undergoing TAVR with a planned access route other than femoral
Faurie et al., 2016^21^	Prospective observational multicenter	87	87	—	—
Tamura et al., 2021^20^	Retrospective, single center	252	204	—	∗∗Patients under 18, not referred for BAV or TAVI; patients receiving RV-TPW pacing
Díaz de la Llera et al., 2018^24^	Prospective, single center	25	25	—	—

Abbreviations: BAV, balloon aortic valvuloplasty; LV, left ventricular; PPM, permanent pacemaker; RV, right ventricular; TAVR, transcatheter aortic valve replacement; TAVI, transcatheter aortic valve implantation; TPW, temporary pacing wire.

**Table 2:** Patient characteristics in the included studies

Study (ref. #)	N	Age (y)	Female sex	Risk score(s)		LVEF (%)	NYHA class			
				EuroSCORE (%)	STS score (%)		I	II	III	IV
Faurie et al., 2019^23^	303	82.94 ± 5.62	75/303 (49.5%)	12.99 ± 10.05	4.85 ± 4.81	59.08 ± 12.37	—	—	—	—
Savvoulidis et al., 2022^27^	1226	82 (77-86)	529/1226 (43.1%)	12.3 (8.44-19.84)	—	**>50**: 859 (70%) **30-49**: 230 (18.8%) **<30**: 137 (11.2%)	156 (12.7%)		1070 (87.3%)	
Hokken et al., 2021^28^	672	80 (74-84)	307/672 (45.7%)	—	—	—	—	—	—	—
Stąpór et al., 2020^25^	143	81 (78-84)	88/143 (61.5%)	3.2 (1.9-5.4)	3.7 (2.7-5.8)	60 (50-65)	5/143 (3.7%)	59/143 (43.4%)	60/143 (44.1%)	12/143 (8.8%)
Kotronias et al., 2019^29^	529	83 (79-86)	239/529 (45.2%)	—	—	—	—	—	—	—
Scarsini et al., 2019^31^	263	83 (79-86)	111/226 (49.1%)	—	—	**>50**: 170 (75%) **<50**: 56 (25%)	6 (0.7%)		220 (97.3%)	
Spaziano et al., 2017^43^	184	83 ± 5.9	88/142 (47.8%)	16.5 ± 11.6	5.3 ± 4.7	—	—	—	—	—
Hilling-Smith et al., 2017^22^	132	83.2	50/132 (37.5%)	—	—	—	—	—	—	—
Faurie et al., 2016^21^	87	85.5 ± 5.3	44/87 (54.3%)	19.7 ± 11.7	—	—	15/87 (18.5%)	4/87 (4.9%)	34/87 (42.0%)	28/87 (34.6%)
Tamura et al., 2021^20^	252	85.1 ± 5.1	152/204 (74.5%)	3.2 (2.1-5.4)	5.8 (4.1-8.0)	64 (59-67)	—	—	—	—
Díaz de la Llera et al., 2018^24^	25	79.2 ± 4.6	—	5.35 ± 3.9	5.81 ± 4.2	—	14/25 (56%)		11/25 (44%)	

Notes. Continuous variables are presented as mean ± SD or median (IQR).

Abbreviations: LVEF, left ventricular ejection fraction; NYHA, New York Heart Association; STS, Society of Thoracic Surgeons.

## Rapid Ventricular Pacing in TAVR

Rapid ventricular pacing was first reported as an essential step in BAV.^32^ It provides assisted cardiac standstill to optimize balloon dilatation and prevent balloon migration during inflation. The technique typically requires either femoral or internal jugular venous access with a ≥5 French (Fr) sheath and a temporary pacing lead delivered to the RV apex.^33^ In TAVR, the role of rapid pacing is more important than in BAV, as it enables accurate valve implantation while minimizing the risk of device embolization.^23^ Rapid pacing is invariably used during balloon-expandable THV deployment; however, it is used more selectively for self-expanding TAVR and often at variable, slower rates. Pacing is essential for balloon postdilatation on all THV devices.

## Complications of Right Ventricular Pacing

Temporary pacemaker leads placed in the RV carry inherent risks including infection, access site-related bleeding, vascular complications, and cardiac perforation with potential tamponade.^15^^,^^34^ Deep venous thrombosis, pulmonary embolism, and pneumothorax, which are access-site-specific, have also been reported.^15^^,^^34^ A recent review by Tjong et al. summarized 32 studies from 1990 to 2019, which included 4546 patients who underwent transvenous temporary pacing for various indications (e.g., atrioventricular block, cardiac arrest, myocardial infarction, and drug toxicity).^15^ The authors reported an overall access-related complication rate of 2.0% during temporary pacing, of which 0.5% was attributable to minor bleeding, 0.5% to unintended arterial puncture, and 0.7% to excessive bleeding. Cardiac tamponade and cardiac perforation occurred in 0.6% and 1.6% of patients, respectively. Fever (>38 °C) and local wound infection were less common, occurring in 0.4 and 1.3%, respectively. Overall, the death rate related to temporary pacing was low (0.2%). Of note, there was a significant decrease in the total complication rates between the 10-year intervals from 1990 to 2019.

Similar complications have been reported with RV pacing during TAVR. However, since the TAVR procedure is different in nature and complexity from temporary pacing for other indications, it is challenging to assess the rates of vascular and structural complications that are directly related to temporary pacing in TAVR. Furthermore, during TAVR, heparin is given to patients to prevent thromboembolic complications, which increase the risk and rates of bleeding and vascular complications. On the other hand, unlike the use of temporary pacing for other acquired bradyarrythmias, the requirement for pacing for TAVR is relatively short, minimizing the duration the wire remains *in situ* and the risk for complications. Reported cardiac tamponade rates with RV pacing during TAVR range between 0.2 and 4.3%,^35^^,^^36^ with the majority (52.9%) attributed to RV perforation by the temporary pacing wire.^36^ The remainder were due to annular rupture or aortic dissection (23.5%) or LV perforation by the stiff guidewire (23.5%). Similarly, in the EASY TAVI randomized trial, the cardiac tamponade rate in the RV pacing group was 2.6%, with half attributed to RV injury caused by the pacing lead.^23^ Importantly, these inherent risks associated with RV pacing remain a concern when bailout from LV pacing due to persistent pacemaker dependence is needed. Of note, in the early days of TAVR, non-balloon-tipped leads were used for rapid pacing, though currently, balloon-tipped leads are more commonly used as this has been shown to be a safer alternative.^35^, ^36^, ^37^, ^38^ Most studies included in the current review of pacing strategies in TAVR utilized balloon-tipped leads (Table 3).

**Table 3:** Procedural characteristics

Study	Valve type				Balloon tipped lead used for RV pacing	Guidewire type				Balloon predilatation		Balloon postdilatation		Rate of pacing (bpm)
	Balloon expandable (Sapien XT/S3/S3 Ultra)		Self-expandable/other (CoreValve, Evolut, Acurate, Lotus)			Amplatz Extra Stiff/Super Stiff	Confida	Safari (Extra Small/Small)	Other (Lunderquist)	LV	RV	LV	RV	
	LV	RV	LV	RV										
Faurie et al., 2019^23^	151/151 (100%)	152/152 (100%)	0/151 (0%)	0/152 (0%)	Yes	128/151 (84.8%)	3/151 (2.0%)	20/151 (13.2%)	0/151 (0%)	14/151 (9.2%)	10/152 (6.6%)	—	—	160-220
Savvoulidis et al., 2022^27^	672/756 (88.9%)	410/470 (87.2%)	84/756 (11.1%)	60/470 (12.8%)	Yes	672/756 (88.9%)	—	85/756 (11.1%)	—	98/756 (12.96%)	207/470 (44.1%)	197 (26.1%)	126 (30.7%)	140-200
Hokken et al., 2021^28^	262/488 (53.7%)	22/45 (48.9%)	226/488 (46.3%)	23/45 (51.1%)	Yes	—	—	—	—	181/488 (37.1%)	15/45 (33.3%)	188/488 (38.5%)	12/45 (26.7%)	—
Stąpór et al., 2020^25^	9/82 (11%)	17/61 (27.9%)	73/82 (89%)	41/61 (67.2%)	No	—	—	—	—	71/82 (86.6%)	41/61 (67.2%)	59/82 (72%)	37/61 (60.7%)	140-180
Kotronias et al., 2019^29^	85/529 (16.0%)	444/529 (84.0%)	—	—	—	—	—	—	—	—	—	—		
Scarsini et al., 2019^31^	37/226 (16.4%)	—	189/226 (83.6%)	—	—	—	—	—	—	121/226 (53.5%)	—	34/226 (15.0%)	—	—
Spaziano et al., 2017^43^	97/142 (52.7%)	87/142 (47.3%)	—	—	—	—	—	—	—	—	—	—		
Hilling-Smith et al., 2017^22^	8/132 (6.1%)	—	124/132 (93.9%)	—	—	—	—	—	—	110/132 (83.3%)	—	—	—	—
Faurie et al., 2016^21^	—	—	—	—	Yes	—	—	—	—	—	—	—	—	160-200
Tamura et al., 2021^20^	79/204 (38.7%)	—	125/204 (61.3%)	—	—	0/204 (0%)	34/204 (16.6%)	163/204 (80%)	7/204 (3.4%)	—	—	—	—	—
Díaz de la Llera et al., 2018^24^	25/25 (100%)	—	0/25 (0%)	—	Yes	—	—	—	—	—	—	—	—	180-240

Abbreviations: LV, left ventricular; RV, right ventricular.

Among patients undergoing temporary pacing for a variety of indications, lead dislodgement and failure of pacing (defined as malpacing or malsensing) were not infrequent (4.6 and 9.5%, respectively).^15^ By comparison, when reported, LV pacing failure (defined as loss-of-capture) in TAVR patients was generally less than 1.0% (Table 4); however, none of the studies reviewed reported rates of RV pacing failure in TAVR patients. Successful pacing was defined as the achievement and maintenance of a systolic blood pressure <60 mm Hg for more than 30 ​seconds without loss-of-capture in two TAVR studies,^23^^,^^25^ with reported rates for LV pacing of 84.9 and 97.6% and a rate of 87.1% for RV pacing.

**Table 4:** Procedural outcomes

Study (ref. #)	Successful THV deployment rate		Crossover to TPW	Bridging TPW d/t conduction disturbances		PPM rate		Loss of capture		Valve dislodgement‡		Valve embolization		Procedure duration (min)		Radiation exposure (mGy)		Cost of LV pacing (compared with RV pacing)
	LV	RV		LV	RV	LV	RV	LV	RV	LV	RV	LV	RV	LV	RV	LV	RV	
Faurie et al., 2019^23^	151/151 (100%)	151/152 (99.3%)	0/151 (0%)	14/151 (9.3%)	38/152 (25.0%)	27/151 (17.9%)	18/152 (11.8%)	—	—	—	—	0/151 (0%)	0/152 (0%)	48.40 ± 16.9	55.60 ± 26.9	355.35 ± 257.88	375.55 ± 255.27	- €528
Savvoulidis et al., 2022^27^	752/756 (99.5%)	465/470 (99.0%)	7/756 (0.9%)	27/756 (3.6%)	20/470 (4.3%)	41/756 (5.4%)	36/470 (7.7%)	—	—	1/756 (0.4%)∗	2/470 (0.4%)∗	1/756 (0.1%)	1/470 (0.2%)	70 (60-80)	80 (70-90)	—	—	- €180
Hokken et al., 2021^28^	—	—	1/488 (0.2%)	24/488 (4.9%)	36/45 (80.0%)	53/488 (10.9%)	21 (46.7%)	1/488 (0.2%)	—	—	—	8/488 (1.6%)	1/45 (2.2%)	55 (43-71)	68 (52-88)	—	—	—
Stąpór et al., 2020^25^	80/82 (97.6%)	57/61 (93.4%)	—	3/82 (3.7%)	4/61 (6.6%)	4/82 (4.9%)	10/61 (16.4%)	2/82 (2.4%)	—	1/82 (1.2%)†	1/61 (1.6%)†	—	—	80 (70-90)	85 (70-95)	575.5 (356.8–974)	437 (265-664)	- €130
Kotronias et al., 2019^29^	—	—	—	—	—	—	—	—	—	—	—	—	—	70 (60-90)	—	—	—	
Scarsini et al., 2019^31^	224/226 (99.1%)	—	2/226 (0.9%)	17/226 (7.5%)	—	30/226 (14%)	—	0/226 (0%)	—	4/226 (1.7%)∗	—	—	—	65 (60-80)	—	1115 (600-1911) G/cm^2^	—	—
Spaziano et al., 2017^43^	142/142 (100%)	—	—	20/142 (14.1%)	19/42 (45.2%)	16/142 (11.3%)	15/42 (35.7%)	—	—	—	—	0/142 (0%)	0/42 (0%)	—	—	—	—	—
Hilling-Smith et al., 2017^22^	120 (90.9%)	—	—	6/132 (4.5%)	—	28/132 (21.2%)	—	0/132 (0%)	—	—	—	0/132 (0%)	—	—	—	—	—	—
Faurie et al., 2016^21^	86/87 (98.9%)	—	—	12/87 (13.8%)	—	14/87 (16.1%)	—	0/87 (0%)	—	1/87 (1.1%)∗	—	—	—	68.7 ± 30.9	—	46.7 ± 38.7 G/cm^2^	—	—
Tamura et al., 2021^20^	202/204 (99.0%)	—	2/204 (1.0%)	29/204 (14.2%)	—	12/87 (5.9%)	—	2/204 (1.0%)	—	2/204 (1.0%)‡	—	—	—	—	—	—	—	—
Díaz de la Llera et al., 2018^24^	25/25 (100%)	—	—	1/25 (4.0%)	—	1/25 (4.0%)	—	0/25 (0%)	—	—	—	0/25 (0%)	—	—	—	—	—	—

Abbreviations: LV, left ventricular; PPM, permanent pacemaker; RV, right ventricular; THV, transcatheter heart valve; TPW, temporary pacing wire.

∗ Malpositioning.

† Displacement.

‡ Migration.

## Direct Left Ventricular Pacing

The feasibility of temporary cardiac pacing without placing a transvenous RV lead has previously been demonstrated in patients with acute coronary syndrome undergoing percutaneous coronary intervention, in whom effective pacing was accomplished through a coronary guide wire.^39^ Similarly, reports have described direct LV pacing through the LV stiff guidewire during BAV.^20^, ^21^, ^22^, ^23^, ^24^, ^25^, ^26^, ^27^, ^28^, ^29^^,^^31^^,^^40^ In TAVR, however, reliable rapid pacing is more critical due to the risk of loss of capture during valve deployment, resulting in valve migration or embolization. We identified 11 studies reporting the use of an LV lead for rapid pacing during valve deployment and meeting criteria for this review (Table 1).

### Technique

Direct LV pacing is most typically performed with the cathode of an external pacemaker attached to the distal end of the LV guidewire via an alligator clip (crocodile clip) or a surgical clamp. The THV, or balloon delivery catheter, then serves as the necessary insulator for the guidewire during LV pacing. The anode is similarly attached either directly to the patient's skin or through a 21 G needle inserted in the subcutaneous tissue around the anesthetized site of the femoral arterial sheath.^23^^,^^25^^,^^31^ Alternatively, other studies have reported the insertion of a guidewire in the inferior vena cava through femoral venous access and with the anode attached to the guidewire.^31^ While less frequently employed, this system facilitates emergent access for RV pacing, if needed, though at the expense of cost, time, and potential access complications.

### Selection of Guidewire for LV Pacing

Although various nondedicated, stiff guidewires have been used for LV pacing during TAVR (Table 3), none of these guidewires have been approved by the United States Food and Drug Administration (FDA) or other regulatory bodies for LV pacing (Supplementary Figure 2). The Amplatz Super Stiff (Boston Scientific, Marlborough, MA, USA), Amplatz Extra Stiff (Cook Medical, Bloomington, IN, USA), and Safari^2^ small/extra small curve (Boston Scientific, Marlborough, MA, USA) were the most reported guidewires used for LV pacing compared to other guidewires such as Lunderquist (Cook Medical, Bloomington, IN, USA) and Confida (Medtronic, Minneapolis, MN, USA) (Table 3). It is worth noting that the efficacy of pacing may vary according to guidewire type. Tamura et al. reported overall successful rapid LV pacing in 99% (202 out of 204) of patients, with the Safari^2^ guidewire being used in the two patients for whom LV pacing could not be achieved. Furthermore, the Confida wire had a significantly lower pacing threshold compared with Safari^2^ (3.0 vs. 5.0 V, *p* < 0.0001) (Table 3), which may be attributed to the fact that the Confida wire tip is more flexible, enabling the wire to fit more easily into the complex shape of the LV.^20^ Nevertheless, it remains somewhat unclear whether these small differences in threshold will translate into clinically meaningful differences.

Recently, two dedicated pacing stiff guidewires, the Wattson wire (Teleflex, Wayne, PA, USA) and the SavvyWire (OpSens Medical, Québec, Canada), were approved by the FDA for rapid LV pacing during TAVR (Supplementary Figure 3). These guidewires were designed with an insulated shaft and exposed wire (on both ends), enabling efficient pacing at relatively lower thresholds in addition to providing the needed support for valve delivery. The Wattson wire is a 280 cm long, 0.035" preshaped stiff guidewire with hydrophilic coating applied on the outer surface of the wire shaft. It enables bipolar pacing since it has positive and negative electrodes that terminate at two different sites on the distal (LV) curve. The proximal tip of the wire is connected to an adapter, which converts it to discrete positive and negative terminal pins that are compatible with a standard external pacemaker. Hensey et al. reported a 100% success rate (defined as successful rapid pacing and valve deployment with no loss of capture) when the Wattson wire was used in 20 patients.^41^ The SavvyWire is also a 280 cm long, 0.035" preshaped stiff guidewire that enables unipolar pacing by connecting the external pacemaker cathode to a specialized noninsulated connection zone on its proximal end while the anode is connected to the patient's skin. The wire also encompasses a pressure sensor at its distal (LV) end that provides real-time hemodynamic measurements displayed on a dedicated screen throughout the procedure. In the first-in-man study, successful pacing (defined as a reduction of at least 50% in systolic blood pressure and/or a systolic pressure value < 60 mmHg) and successful valve deployment were achieved in all 20 patients without any loss of capture events.^42^ Notably, both wires are longer than most other commercially available guidewires, which facilitates full removal of the valve delivery system without disconnecting the wire from the pacemaker, thus ensuring continued pacing in case backup RV pacing is required for intraprocedural bradyarrythmias.^42^

### Procedural Success

Procedural success is a critical outcome to determine whether direct LV pacing is comparable to RV stimulation in TAVR; however, the definitions across studies varied (Table 3). Regardless of the definition used, all studies reported high success rates with LV pacing, ranging between 90.9% and 100% (Table 4). Of particular note, in the studies comparing the two pacing methods, procedural success was comparable between RV and LV pacing. Importantly, valve dislodgment and embolization were very infrequent (0.4%-2.2%) in all studies (Table 4). Crossover rates due to pacing failure (defined as failure to deliver consistent and reliable capture for rapid pacing) in the LV group were not reported in all studies, but in those that did, rates were below 1.0% (Table 4).^23^^,^^27^^,^^31^ Regarding the need for temporary pacing after TAVR due to new-onset conduction disturbances, all three studies comparing pacing methods reported that less than 10% of patients needed temporary RV pacing. Interestingly, in the EASY TAVI trial,^23^ only 9.3% of patients in the LV cohort required an RV lead in situ after TAVR, compared with 25.0% of the patients in the RV cohort (Table 4). This implies that physicians tend to be more “conservative” and keep the transvenous pacer in situ in cases in which it is already positioned in the RV, a difference with potential clinical implications. In all cases that required cardiac pacing immediately postimplant, LV pacing provided a stable transition to allow for the introduction of an RV lead.

## Other Procedural Outcomes

The potential benefits of LV pacing include reduced procedure duration and cost. The results of studies reporting on these metrics are shown in Table 4. The only prospective, multicenter, randomized trial was EASY TAVI.^23^ In this study, there were no exclusion criteria for underlying conduction system disease. The primary endpoint, procedure duration, defined as the time from first vascular puncture until withdrawal of the last vascular access sheath, was found to be lower in the LV stimulation group as compared with the RV stimulation group (48.4 ± 16.9 ​minutes vs. 55.6 ± 26.9 ​minutes; *p* = 0.0013, respectively). Similarly, all other studies comparing procedure duration between pacing groups also reported it to be significantly shorter with LV pacing. Reduced procedure time was associated with decreased radiation exposure in most studies (Table 4). Only one study indicated a numerical increase in radiation dose in the LV cohort despite a shorter procedure duration, which the authors attributed to the need for more frequent predilatation in LV guidewire pacing rather than the pacing method itself.^25^

LV pacing was not only associated with shorter procedures and generally less radiation, but it also led to reductions in cost. The EASY TAVI trial^23^ determined that although there was no significant difference in the length of hospital stay between groups, both procedural and overall costs at 30 days were significantly lower in the LV stimulation group when compared with the RV stimulation group. Similarly, other nonrandomized studies reported TAVR with LV pacing to be associated with lower costs (Table 4).^25^^,^^27^

### Clinical Outcomes

Major adverse cardiovascular events were the primary endpoint of two studies, and one of these studies found them to be significantly less common with LV pacing (Table 5). In studies that reported short-term mortality (in-hospital and 30 days postprocedure), rates were lower, though not statistically significant, in the LV vs. RV group. Major vascular complications were also less frequent in the LV pacing cohorts, while minor vascular complications and bleeding complications were comparable between groups. Overall, tamponade rates were low across all studies, regardless of pacing strategy (Table 5). EASY TAVI, the only randomized trial, reported a lower, though not statistically significant, rate of tamponade in the RV pacing cohort (1.3 vs. 2.6%; *p* = 0.68).^23^ In the largest retrospective analysis of 1226 patients, of which 756 (61.7%) underwent TAVR with LV pacing,^27^ the rate of tamponade was numerically lower with LV vs. RV pacing though the difference did not reach statistical significance (1.5 vs. 2.3%; *p* = 0.27). Expectedly, no events of tamponade were attributed to RV perforation in the LV pacing group, compared with 6 events (2.3%) in the RV pacing group (*p* < 0.005).^27^ Similarly, Kotronias et al.^29^ reported that LV guidewire pacing significantly decreased the rate of pericardial effusion and cardiac tamponade (*p* = 0.021), and Scarsini et al.^31^ determined only 3 out of 226 patients in the LV cohort (1.3%) had tamponade that was attributed to annular rupture (Table 5).

**Table 5:** Outcomes

Study (ref. #)	Primary endpoints	Primary endpoint rate			Death				Cardiac tamponade				Pericardial effusion		Bleeding complications		Vascular complications			
					In-hospital		30-day		Annular tear/LV-free wall rupture		RV perforation by TPW						Major		Minor	
		LV	RV	*p* value																
					LV	RV	LV	RV	LV	RV	LV	RV	LV	RV	LV	RV	LV	RV	LV	RV
Faurie et al., 2019^23^	Procedure duration (time from the first vascular puncture until withdrawal of the last vascular access sheath)	48.4 ± 16.9 ​min	55.6 ± 26.9 ​min	*p* = 0.0013	—	—	4/151 (2.6%)	6/152 (3.9%)	2/151 (1.3%)	2/152 (1.3%)	0/151 (0%)	2/152 (1.3%)	—	—	7/151 (4.6%)	7/152 (4.6%)	2/150 (1.3%)	3/152 (2.0%)	6/150 (4.0%)	7/152 (4.6%)
Savvoulidis et al., 2022^27^	Rate of tamponade	1.50%	2.30%	*p* = 0.27	12/756 (1.6%)	17/470 (3.6%)	—	—	11/756 (1.5%)	5/470 (1.1%)	0/756 (0%)	6/470 (1.3%)	—	—	5/756 (0.7%)	6/470 (1.3%)	5/756 (0.7%)	9/470 (1.9%)	21/756 (2.8%)	6/470 (1.3%)
Hokken et al., 2021^28^	RV pacemaker insertion for failed LV pacing (bailout) or occurrence of high-degree blocks mandating continuous pacing	24/488 (4.9%)	—	—	—	—	11/488 (2.3%)	2/45 (4.4%)	—	—	—	—	—	—	12/488 (2.5%)	1/45 (2.2%)	29/488 (5.9%)	1/45 (2.2%)	41/488 (8.4%)	3/45 (6.7%)
Stąpór et al., 2020^25^	MACE (death, stroke, venous puncture related complications, and cardiac tamponade)	4/82 (4.9%)	7/61 (11.5%)	*p* = 0.13	1/82 (1.2%)	1/61 (1.6%)	—	—	**LV:** 0/82 (0%) **RV:** 2/61 (3.3%)∗				16/82 (19.5%)	4/61 (6.6%)	—	—	0/82 (0%)	3/61 (4.9%)	5/82 (6.1%)	4/61 (6.6%)
Kotronias et al., 2019^29^	A hierarchic composite of in-hospital mortality, pericardial effusion/cardiac tamponade, major bleeding, and vascular access complications.	OR (LV vs. RV pacing) = 0.19, CI: 0.05–0.66		*p* = 0.009	**In-hospital:** 11/529 (2%)				19/529 (3.6%); **OR** (LV vs. RV Pacing) = 0.24, CI: 0.07-0.83, *p* = 0.021				—	—	17/529 (3.2%)		**Major:** 19/529 (3.6%) **Minor: 25/529 (4.7%) OR** (LV vs. RV Pacing) = 0.33, CI: 0.11-0.94, *p* = 0.38			
Scarsini et al., 2019^31^	RV pacemaker insertion for failed LV pacing (bailout)	224/226 (99.0%)	—	—	7/226 (3.1%)	—	—	—	3/226 (1.3%)	—	0/226 (0%)	—	3/226 (1.3%)	—	—	—	**Major and Minor (LV):** 6/226 (2.7%)			
Spaziano et al., 2017^43^	Success rate of LV pacing	142/142 (100%)	—	—	—	—	—	—	—	—	—	—	—	—	—	—	—	—	—	—
Hilling-Smith et al., 2017^22^	Success rate of LV pacing without the need for TPW	132/132 (100%)	—	—	—	—	—	—	**LV:** 0/132 (0.0%)∗				—	—	—	—	—	—	—	—
Faurie et al., 2016^21^	Success rate of LV pacing	87/87 (100%)	—	—	4/87 (4.6%)	—	—	—	**LV:** 1/87 (1.1%)∗				—	—	—	—	**Major and Minor (LV):** 10/87 (11.5%)			
Tamura et al., 2021.^20^	Success rate of LV pacing	202/204 (99.0%)	—	—	2/204 (1.0%)	—	—	—	2/204 (1.0%)	—	1/204 (0.5%)	—	—	—	—	—	—	—	—	—
Díaz de la Llera et al., 2018^24^	Success rate of LV pacing	25/25 (100%)	—	—	—	—	1/25 (4.0%)	—	**LV**: 0/25 (0%)∗				—	—	—	—	**Major and Minor (LV):** 1/25 (4.0%)			

Abbreviations: LV, left ventricular; MACE, major adverse cardiovascular events; OR, odds ratio; RV, right ventricular; TPW, temporary pacing wire.

∗ Etiology of tamponade is not reported.

The frequency of permanent pacemaker (PPM) implantation post-TAVR ranged from 4.0 to 46.7% across the various studies (Table 4). Notably, EASY TAVI^23^ reported numerically higher 30-day PPM rates with LV pacing (17.9% vs. 11.8%, *p* = 0.14), while the other nonrandomized studies tended to show higher PPM rates in the RV pacing group. Of note, Hokken et al.^28^ and Spaziano et al.^43^ reported extraordinarily high rates of PPM placement for their RV cohorts (46.7% and 35.7%, respectively). Indeed, both of these small studies included patients with pre-existing conduction system disease in the RV cohort but excluded them from the LV cohort. This selection bias may explain why rates of PPM are directionally opposed in the randomized and nonrandomized studies.

Collectively, these studies suggest that direct LV pacing is feasible and may be a beneficial strategy for rapid pacing in TAVR, as it may be associated with similar procedural success rates, and decreased rates of complications related to RV pacing, procedure duration, and cost.

### Proposed Strategy for Rapid Pacing During TAVR

**Figure 1:**
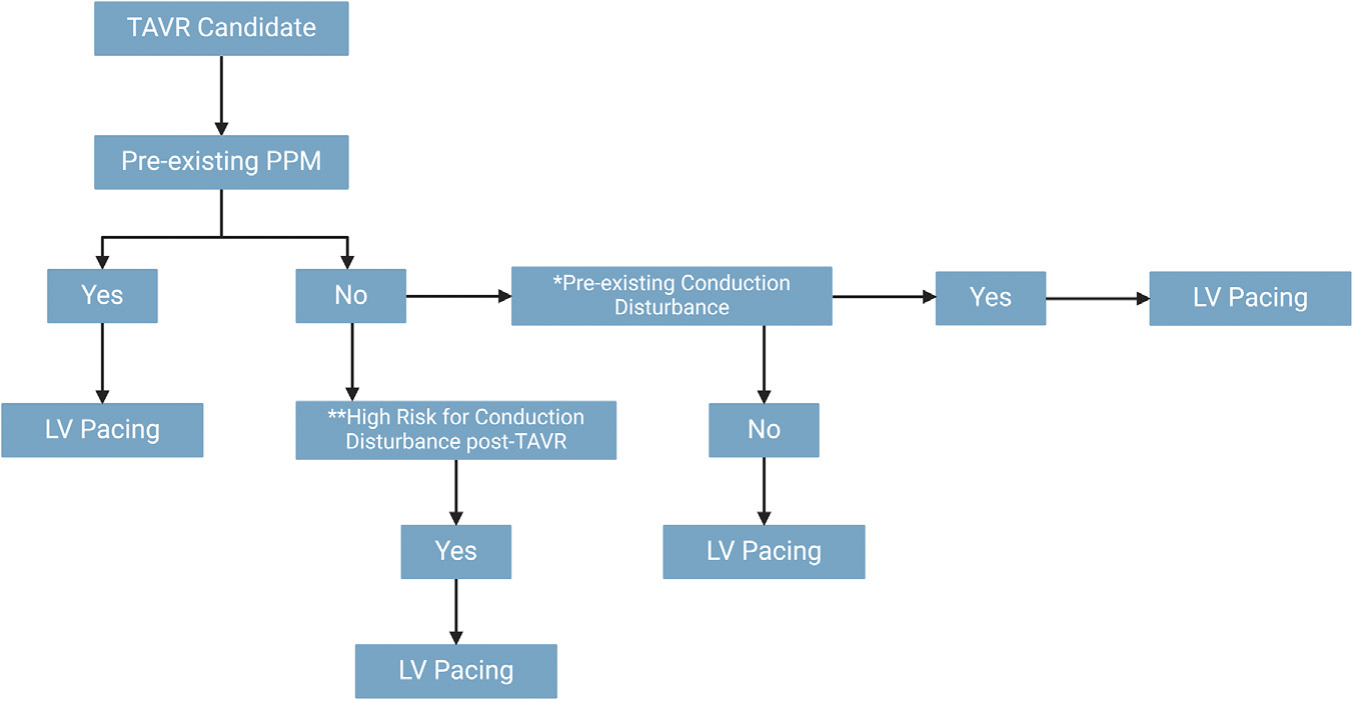
**Algorithm Proposal for Temporary Pacing in TAVR**. Adapted from Rodés-Cabau et al.^44^ and Blusztein et al.^45^ ∗RBBB, LBBB, first-degree AVB, LAHB, IVCD with QRS ≥120, or any combination thereof. ∗∗Anatomical features (severity of valvular/annular/LVOT calcification, length of membranous septum); procedural characteristics (valve type [SEV vs. BEV], planned depth of implantation relative to the membranous septum length, planned degree of oversizing) Abbreviations: AVB, atrioventricular block; BEV, balloon-expandable valve; IVCD, intraventricular conduction delay; LAHB, left anterior hemiblock; LBBB, left bundle branch block; LV, left ventricular; LVOT, left ventricular outflow tract; PPM, permanent pacemaker; RBBB, right bundle branch block; RV, right ventricular; SEV, self-expandable valve; TAVR, transcatheter aortic valve replacement.

Direct LV pacing is a comparable alternative for most patients undergoing TAVR. However, for those with increased risk for conduction disturbances postprocedure and in whom a temporary pacing wire is expected to be kept in place (i.e., patients with pre-existing right bundle branch block, especially in the presence of other conduction disturbances), RV pacing may be preferred. Operator experience as well as patient characteristics (pre-existing conduction disturbances, anatomical features, and procedural characteristics) must be taken into account to determine the most appropriate pacing strategy, as suggested in Figure 1.

## Recommendations and Future Directions

• LV pacing may be considered the default pacing method for currently indicated TAVR.
• Standard stiff guidewires, though not FDA-approved for this purpose, are safe and efficacious, and two-purpose-designed LV guidewires are now approved and available.
• Prospective, randomized controlled trials are required to compare the outcomes of LV vs. RV pacing.

## Conclusions

Review of the current literature suggests that direct LV guidewire pacing is comparable in efficacy to RV pacing with possibly improved safety and overall clinical outcomes attributed mainly to lower rates of cardiac perforation and tamponade related to temporary RV pacing wires. In addition, shorter procedural times, lower radiation, and reduced costs may be anticipated. Although LV pacing can be readily performed using the stiff wire, it is reasonable to assume that there is a learning curve associated with LV pacing during TAVR that operators should be aware of. Special attention must be paid to the position of the stiff wire in the LV before valve deployment, which might be important in order to prevent loss of capture during this critical step. Large, adequately designed, and powered prospective trials are necessary to provide more definitive answers on this topic.

## Funding

This research did not receive any specific grants from funding agencies in the public, commercial, or not-for-profit sectors.

## Review Statement

Full responsibility for the editorial process for this article was delegated to guest editor Francesco Maisano, MD.

## Disclosure Statement

T. Nazif has performed consulting for or received honoraria from Edwards Lifesciences, Medtronic, Venus Medtech, and Boston Scientific. M. Leon has received instructional research grants from Abbott, Boston Scientific, Edwards Lifesciences, and Medtronic. All other authors have reported that they have no relationships relevant to the contents of this paper to disclose.
