# Supplementary material for: Contemporary Review of the Methods for Rapid Ventricular Pacing During Transcatheter Aortic Valve Replacement
Source: Struct Heart. 2024 Jun 13;9(2):100306. doi: 10.1016/j.shj.2024.100306 (PMC11925033; doi:10.1016/j.shj.2024.100306)
Supplement: Supplementary Appendix [file mmc1.docx]

**Supplementary Figures**

**Supplementary Figure 1.** Preferred Reporting Items for Systematic Reviews and Meta-Analyses (PRISMA) flowchart of our analysis.

Records removed before screening:

Duplicate records removed (n=586)

Records removed for other reasons (n=0)

Records identified from:

PubMed (n=445)

EMBASE (n=426)

Web of Science (n=13)

**Identification**

Records screened

(n=298)

Records excluded* (n=278)

Reports sought for retrieval

(n=27)

Reports not retrieved

(n=0)

**Screening**

Reports excluded:

Small (<20 patients) sample size (n=4)

Conducted in non-humans (n=1)

No mention of complications (n=4)

Reports assessed for eligibility

(n=27)

­

Studies included in review

(n=11)

**Included**

*non-human, unrelated to TAVR, unrelated to pacing, not published in English

**Supplementary Figure 2.** Non-dedicated wires for LV pacing. Adapted with permission from Tamura et al.^20^


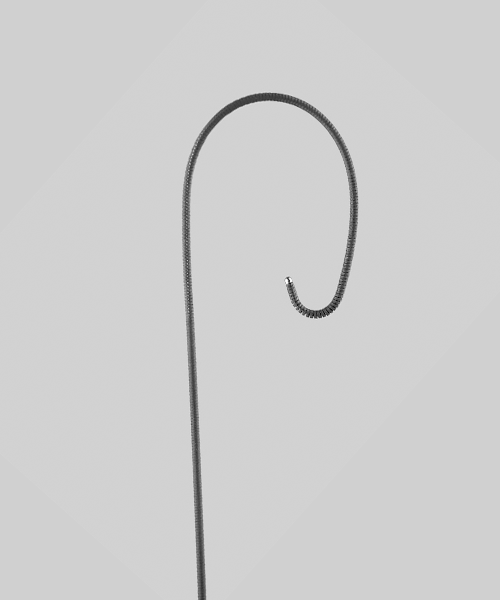

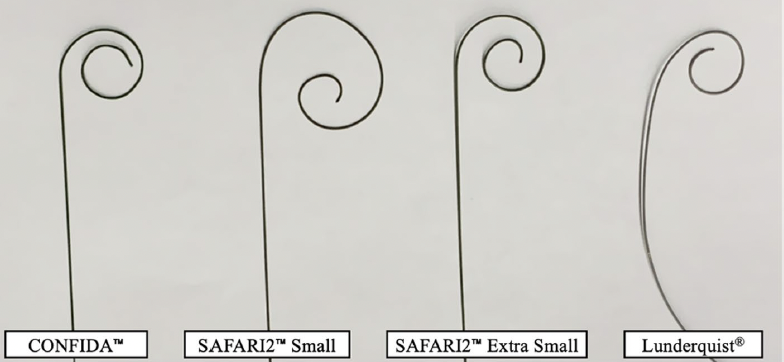


Amplatz^™^


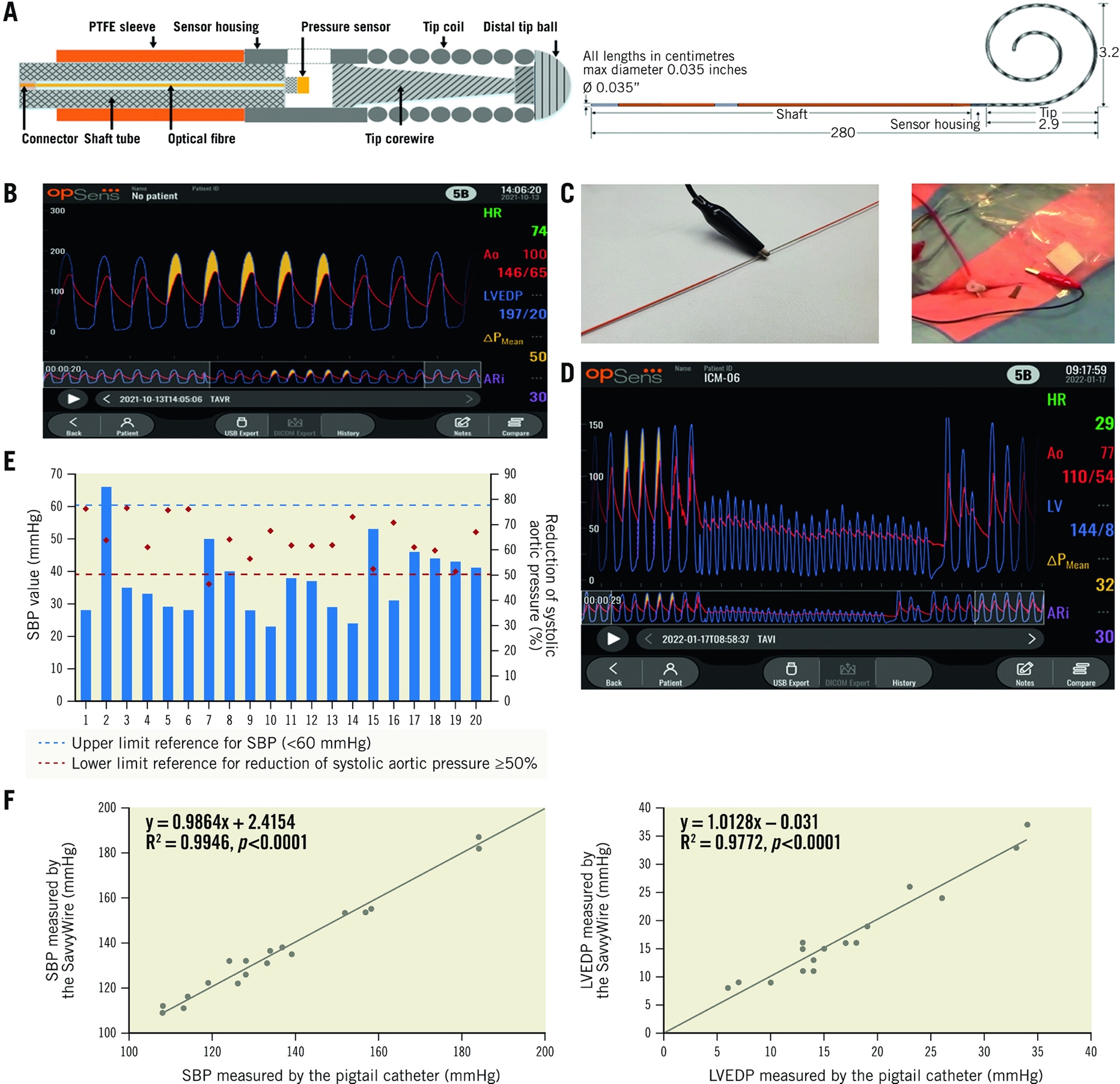
**Supplementary Figure 3.** Dedicated wires for LV pacing. A. Wattson wire. Red arrows indicate exposed electrodes on the Wattson wire.^41^ B. SavvyWire.^42^

SavvyWire

**B**

**A**


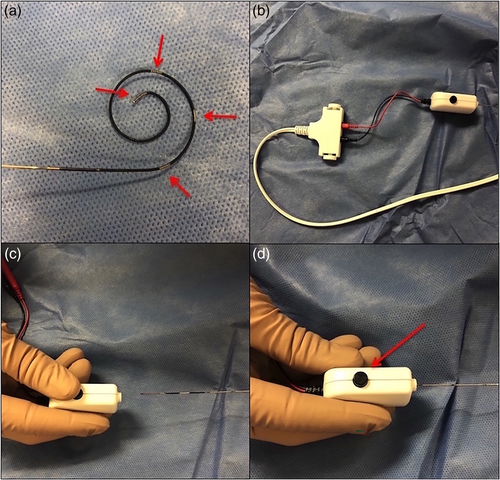


Wattson Wire
